# Manipulation under Anesthesia versus Non-Surgical Treatment for Patients with Frozen Shoulder Contracture Syndrome: A Systematic Review

**DOI:** 10.3390/ijerph19159715

**Published:** 2022-08-07

**Authors:** Mattia Salomon, Chiara Pastore, Filippo Maselli, Mauro Di Bari, Raffaello Pellegrino, Fabrizio Brindisino

**Affiliations:** 1 Department of Clinical Science and Translational Medicine, University of Roma “Tor Vergata”, 00133 Rome, Italy; 2 Department of Human Neurosciences, University of Roma “Sapienza”, 00185 Rome, Italy; 3 Research Unit of Medicine of Aging, Department of Clinical and Experimental Medicine, University of Florence, 50121 Florence, Italy; 4 Unit of Geriatrics—Geriatrics Intensive Care Unit, Department of Medicine and Geriatrics, “Careggi Hospital”, 50134 Florence, Italy; 5 Antalgic Mini-Invasive and Rehab-Outpatients Unit, Department of Medicine and Aging Sciences, University “G. D’Annunzio” Chieti-Pescara, 66100 Chieti, Italy; 6 Department of Scientific Research, Campus Ludes, Off-Campus Semmelweis University, 6912 Lugano, Switzerland; 7 Department of Medicine and Health Science “Vincenzo Tiberio”, University of Molise, “Cardarelli Hospital”, 86100 Campobasso, Italy

**Keywords:** adhesive capsulitis, frozen shoulder, frozen shoulder contracture syndrome, manipulation under anesthesia, physiotherapy, systematic review

## Abstract

Purpose: To investigate the efficacy of manipulation under anesthesia (MUA) compared to other non-surgical therapeutic strategies for patients with frozen shoulder contracture syndrome (FSCS). Methods: A systematic review of literature was conducted. A literature search was performed in MEDLINE, EMBASE, PEDro, Cochrane Central Library and Scopus. Only randomized controlled trials were included and assessed for critical appraisal through the Cochrane Collaborations tools. Results: Five randomized controlled trials were included. The overall risk of bias (RoB) was high in 4 out of 5 of the included studies. MUA was found to be not superior in terms of reduction of pain and improvement of function when compared to cortisone injections with hydrodilatation (mean regression coefficient MUA −2.77 vs. injection −2.75; 95% CI (−1.11 to 1.15)) and home exercise (mean difference 95% CI: 0.2 (−0.64 to 1.02)) in the short term (3 months), and cortisone injections with hydrodilatation (mean regression coefficient MUA 3.13 vs. injection 3.23; 95% CI (−0.90 to 1.11)) in the long term (>6 months). Moreover, if compared to structured physiotherapy, MUA highlighted a higher Oxford Shoulder Score at final 1-year follow up (mean difference 95% CI: 1.05 (−1.28 to 3.39); *p* = 0.38). Similar results were obtained for disability, with statistically no significant long-term (>12 months) differences between MUA and home exercise (mean difference 95% CI: 0 (−3.2 to 3.2)) or structured physiotherapy (mean difference 95% CI: −0.50 (−5.70 to 4.70); *p* = 0.85)). Only two trials reported adverse events. Conclusions: This review suggested that limited and inconsistent evidence currently exists on the efficacy of MUA compared to other non-surgical strategies in the management of patients with FSCS. Future research should focus on clinical trials with higher methodological quality.

## 1. Introduction

Frozen Shoulder Contracture Syndrome (FSCS) [1] is a non-traumatic condition of uncertain etiology, characterized by the gradual onset of pain and significant restriction of shoulder movements, both active and passive, which occurs in the absence of known intrinsic shoulder dysfunctions and with normal radiographic features [2]. FSCS is typically described as a three-stage 12- to 18-month self-limited process [3,4] with symptoms that may persist for years [5] and can be seriously disruptive: residual pain and functional limitations are reported in the long term in 6–16% of patients on average for 4.4 years (range, 2–20 years), with consequent significant impacts on almost every aspect of daily activities and quality of life [6]. For this reason, the treatment of FCSC should focus on limiting symptoms and shortening the duration of disabilities [7].

However, the available evidence on treatment efficacy does not suggest a consensus [8,9,10]: many controversies exist on which should be considered the best treatment option [11], making the management challenging for clinicians [12]. Different types of treatment, consisting of intra-articular corticosteroid injections [13,14,15] and physiotherapy, including manual therapy [16,17], exercise [18] or arthrographic joint distension (also known as hydrodilatation) [19,20], are described in the literature. These non-surgical treatments are considered appropriate for most patients and sufficient to alleviate symptoms in most cases [10], but uncertainties and discussions regarding their long-term efficacy still persist [9,21,22].

Conversely, among surgical procedures, manipulation under anesthesia (MUA) is a widely used procedure, both as a stand-alone treatment option [21,22] and combined with other interventions [23,24]. MUA is an invasive procedure that has been claimed to rapidly reduce symptoms, restore range of motion (ROM) and reduce mean recovery time [25], especially for “resolution” phase or resistant FSCS [21,22]. In fact, key indicators to advocate for surgical interventions (such as MUA or arthroscopic capsular release) are failure of previous conservative treatment, time relapse and symptoms from the onset, although there is some disagreement about the role of pain as a predictor for surgery [26]. Different manipulation techniques are described in the literature, while general principles are patient’s analgesia and passive shoulder mobilization along different combinations of directions, causing the capsule to stretch or tear. However, the main disadvantage of MUA procedures is that uncontrolled manipulation could cause structural damages in the glenohumeral joint and its surrounding soft tissues [27,28]. Even with a low complication rate of 0.4% [27], serious post-surgery complications are reported [29,30]. As a result, the applicability of MUA can be considered controversial [7,10] and a substantial lack of agreement among orthopedic surgeons about MUA procedures exists [31]. To date, almost exclusively observational studies have been used to guide clinical practice, whereas no secondary research publications have been produced focusing exclusively on MUA.

For this reason, the aim of this systematic review (SR) is to investigate the effectiveness and safety of MUA compared to non-surgical strategies among patients with FSCS.

## 2. Materials and Methods

### 2.1. Data Sources and Searches

The Preferred Reporting Items for Systematic Review and Meta-Analyses (PRISMA) statement [32] was used as a guide for the reporting of this SR. The Cochrane Handbook for Systematic Reviews of Intervention [33] was followed as the methodological guidance. The SR protocol was registered in the PROSPERO database (protocol number: CRD42020155343).

An electronic literature search was conducted in the following databases: MEDLINE through PubMed, EMBASE, Physiotherapy Evidence Database (PEDro), Cochrane Central Library and Scopus. Searches were conducted from studies published up to 31 January 2022. Studies published in English, Italian and German were eligible for inclusion. In addition, other potentially relevant studies were searched in gray literature sources (Google Scholar, conference proceedings, theses, conference reports, direct contact with clinicians and experts, books). Reference lists of all eligible articles identified from the search strategy were also screened to identify any further studies for inclusion.

Specific search strategies were created for each database (Table 1 and Supplementary Materials, Table S1).

### 2.2. Study Selection Criteria

#### 2.2.1. Population

The search was limited only to studies with subjects who were diagnosed with primary and idiopathic FSCS. No sex and age restrictions were applied.

Patients diagnosed with fractures, dislocations (acute or recurrent), complete or partial lesions of the rotator cuff, surgical interventions, infections, tumors and inflammatory or systemic diseases (except for diabetes and thyroid disorders) were excluded.

#### 2.2.2. Intervention and Comparator

Studies in which MUA was compared to one or more non-surgical treatment strategies (in detail, physiotherapy, manual therapy, exercise, stretching, injections and/or other conservative strategies cited in the available literature) were included. In addition, studies that reported exclusively nerve or plexus block procedures (without MUA) or MUA combined in the same group with other treatment procedures were excluded.

#### 2.2.3. Outcomes

We restricted the SR to studies that considered the following outcomes: pain intensity, function, ROM, strength of shoulder muscles, return to activities, return to work, disability, presence of adverse events and other outcomes (health-related quality of life, perceived degree of satisfaction by the patient and/or clinician, perceived quality of treatment).

#### 2.2.4. Study Design

Only randomized controlled trials (RCTs) were included, as recommended by the Cochrane Collaboration [34,35].

### 2.3. Data Extraction

Search results were managed through EndNote X9 (Clarivate Analytics, Philadelphia, PA, USA). Duplicates were automatically removed. Data selection and collection processes were conducted using Rayyan QCRI online software [36] by two independent authors (M.S. and C.P.). A third blinded author (F.B.) was involved in case of disagreement. The selection was made by reading the title, abstract and subsequently the full text, assessing its congruency with the declared inclusion/exclusion criteria.

Cohen’s kappa (K) statistic was used to quantify the inter-rater agreement between the two authors (M.S. and C.P.) and interpreted according to Altman’s definition [37].

A planned standardized Excel spreadsheet was used to extract data (type of study, patients’ characteristics, type of intervention, outcomes, follow-up times, adverse events and other relevant information).

### 2.4. Methodological Quality and Risk of Bias Assessment

Two independent authors (M.S. and F.M.) were involved in methodological quality assessment, using the revised Cochrane risk of bias tool for randomized trials (RoB 2.0 tool) for the RCTs [38]. RoB 2.0 analysis was graphically summarized through the RoB graph and the RoB diagram obtained with the ROBVIS Tool [39].

Evaluations of each author were compared; disagreements between reviewers in data extraction and assessments of RoB or quality of evidence were resolved by third-party blinded adjudication (F.B.).

### 2.5. Data Synthesis and Analysis

Meta-analysis was not performed because of considerable heterogeneity regarding patient selection criteria, types of intervention, control groups and outcome measures used within the included studies. Therefore, only a qualitative synthesis with a summary of the evidence available was performed.

## 3. Results

### 3.1. Study Selection and Data Extraction

The search strategies retrieved 1655 articles. After removing 748 duplicates, the remaining 907 articles were independently screened by title and abstract and a further 843 records were excluded.

Inter-rater agreement after title and abstract reading (K = 0.78) was good and moderate for full-text selection (K = 0.59). Disagreements for seven studies were solved by the third independent author (F.B.), which achieved consensus among researchers.

**Figure 1:**
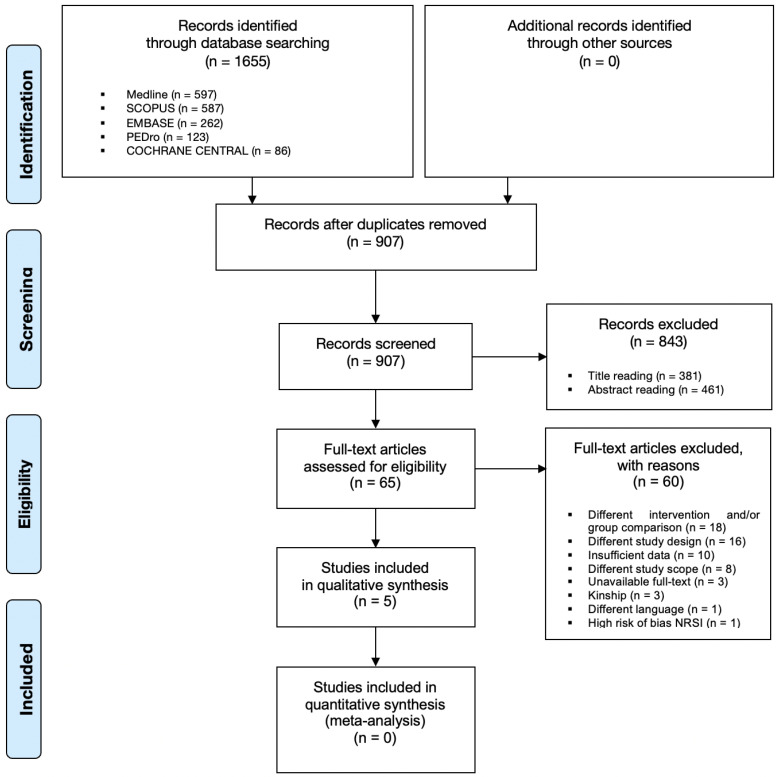
Prisma flowchart for search strategy results.

After reading the full texts, five RCTs [40,41,42,43,44] of the remaining 65 published articles were eligible, and therefore selected for the review. A detailed selection process is shown in the PRISMA flowchart (Figure 1).

Specific reasons for exclusion are listed, as regards the RCTs (Supplementary Materials, Table S2 and S3). Two authors (M.S. and C.P.) independently extracted the data following the study protocol.

The main data, characteristics and results of the studies included were extracted and are listed and are summarized in Table 2.

### 3.2. Methodological Quality and Risk of Bias Assessment

The five eligible RCTs [40,41,42,43,44] received quality assessment: no differences resulted in the attribution of RoB judgement by the two blind reviewers (M.S. and F.M.).

In two RCTs [41,44], the methods of randomization and blinding were adequately described, whereas in the remaining three studies [42,44,45], the presence of bias due to randomization could not be excluded.

The RoB due to deviation from the intended intervention was high in three RCTs [40,41,42]: awareness of treatment procedures was unclear in most cases. Conversely, the RoB related to missing data was judged as low in four studies [40,42,43,44] (which used an intention-to-treat analysis) and as high in the last one [41]. A high RoB in measurement of outcomes was reported in two studies [40,44], as blinding of the outcome assessor was uncertain [40] or not feasible [44]. Only two other RCTs [41,42] obtained a low RoB rating from both evaluators for this domain, while study [43] showed some concerns in domain number 4.

Finally, in four studies [40,41,42,43], a judgment of “some concerns” was expressed for the selection bias of the results reported; in three [41,42,43] of the four RCTs, some inconsistencies and discrepancies were identified with respect to what was stated in the protocol (mostly incomplete reported measures data), while, for one study [40], it was not possible to consult the protocol entirely. In the one remaining RCT [44], RoB was judged low, due to its analyzed results in accordance with a pre-specified analysis plan.

Overall, four out of five RCTs were judged to have a high RoB [40,41,42,44], while the last one was judged as having “some concerns” [43].

**Figure 2:**
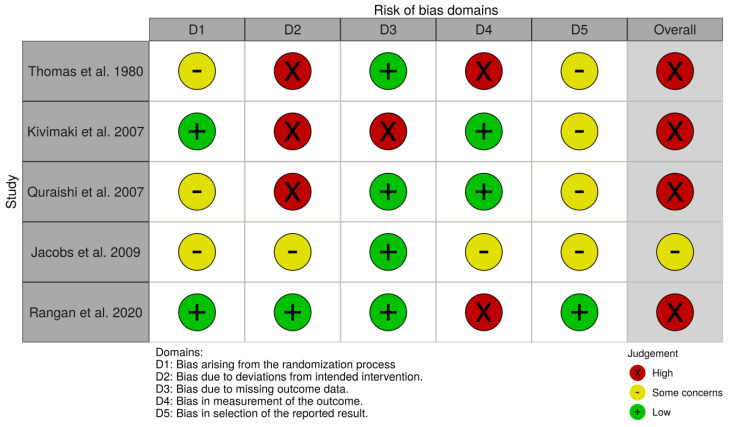
Risk of bias graph for RCTs [42,43,44,45,46].

**Figure 3:**
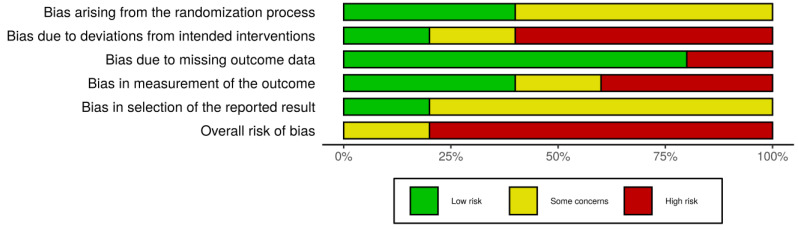
Risk of bias diagram for RCTs.

The evaluation of the methodological quality of the studies included is shown in Figure 2 and Figure 3.

### 3.3. Summary of Evidence

Overall, 544 patients were included. The sample size for each study ranged from 30 patients [40] to 300 patients [44]. The proportion of women compared to men was reported in three studies [43,45,46], ranging from 64% [44] to 80% [43] for the MUA group and from 54% [43] to 67% [44] for the conservative strategies group, respectively.

Patients’ mean age reported in all the studies included [40,41,42,43,44] ranged from 38 to 76 years, with an average value ranging from 53.0 [41] to 59.3 years [40] in the MUA group, and from 53.0 [41] to 57.9 years [40] in the conservative strategies group.

The applied non-surgical therapeutic options for the conservative strategies group were as follows: steroid and anesthetic intra-articular injections (SJ) [40], steroid and anesthetic intra-articular injections associated with hydrodilatation (SJHD) [43] or the hydrodilatation alone (HD) [42], steroid and early structured physiotherapy (SPT) [44] or home exercise (HE) [41].

#### 3.3.1. Pain

All studies considered pain as an outcome to describe the efficacy of the intervention [40,41,42,43,44]. Four [40,41,42,43] out of five RCTs assessed pain through the visual analogue scale (VAS) [45]. Only one study [44] adopted a numeric rating scale (NRS) for pain [46].

Three [42,43,45] out of five RCTs showed no statistically significant difference between the MUA group and SJ group at 1 month [40] (*n* = 12, 80% vs. *n* = 7, 47%), between the MUA group and HE group at 6-week follow-up [41] (mean difference (MD) 95% CI: 0.2 (−0.64 to 1.02)) and between MUA and SJHD groups at 16-week follow-up [43] (mean regression coefficient MUA −2.77 vs. SJHD −2.75; 95% CI (−1.11 to 1.15)).

Furthermore, when the MUA group was compared to the HE group [41], moderate changes in pain intensity at mid-term and long-term follow-ups were detected, with no statically significant difference, both at 3-month follow-up (MD 95% CI: 0.2 (−1.06 to 1.10)), 6-month follow-up (MD 95% CI: −0.8 (−1.8 to 0.2)) and at final 1-year follow-up (MD 95% CI: −0.7 (−1.8 to 0.4)). Overlapping results were highlighted by comparison with the MUA group and SPT group [44]: no statically significant differences between groups were found at 3-month follow-up (MD 95% CI: 0.43 (−0.17 to 1.03)), at 6-month follow-up (MD 95% CI: −0.19 (−0.78 to 0.43)) and at final 1-year follow-up (MD 95% CI: −0.08 (−0.66 to 0.50)).

According solely to one study [42], pain scores measured in the HD group were significantly better than in the MUA group at the final 6-month follow-up (Mann–Whitney test, *p* < 0.0001).

#### 3.3.2. Function

Regarding shoulder function, three RCTs [42,43,44] reported questionable results. The Oxford Shoulder Score (OSS) [47,48], adopted in one RCT [44], showed a satisfying improvement for both groups, with a median overall score of 43 (out of 48 points, where a higher score corresponds to worst function). Nevertheless, no statistically significant differences between the MUA group and SPT group with respect to the decrease in OSS were found at 3 months (MD 95% CI: −1.36 (−3.70 to 0.98), at 6 months (MD 95% CI: 2.15 (−0.12 to 4.42) and at final 1-year follow-up (MD 95% CI: 1.05 (−1.28 to 3.39), respectively. The MUA group had higher mean OSS than the SPT group, but mean estimates were less than the minimal clinically important difference [49].

Similarly, the Constant–Murley Shoulder Function Assessment Score (CS) [50] resulted in conflicting evidence. One study [43] highlighted no statistically significant difference in the increase in CS score between MUA and SJHD, expressed as regression coefficients over time (mean regression coefficient MUA 3.13 vs. SJHD 3.23; 95% CI (−0.90 to 1.11)). Differently, the HD group showed favorable statistically significant changes in the CS score when compared to the MUA group, at a 6-month follow-up (Mann–Whitney test, *p* = 0.02) [42].

#### 3.3.3. Range of Motion

As far as ROM is concerned, one study [40] described a complete 3-month recovery in active ROM in favor of the MUA group: 12 patients (80%) compared to 7 patients (47%) in the SJ group. Similarly, another study [41] reported a statistically significant difference for shoulder passive ROM in forward flexion at 3 months in the MUA group when compared with the HE group (144° and 136°, respectively; MD 95% CI: 8° (0° to 16°)).

Conversely, the MUA group, compared to the HD group [42], showed no statistically significant differences at 6 months for abduction, anterior flexion, external rotation or internal rotation.

#### 3.3.4. Disability

To describe the efficacy of the intervention, disability outcome measures were reported in three RCTs [42,43,46]. One study [40] compared disability with a four-point scale (scored as “0” if worse, “1” if no change, “2” if improved and “3” if cured): seven patients improved completely in the MUA group and only two in the SJ group (47% and 13%, respectively) at a 3-month follow-up.

As previously described for pain, no statistically significant differences were identified in two RCTs [41,44] at mid-term and long-term follow-up periods (6 months and 1 year period of follow-up). The MUA group was found to be not superior in decreasing disability when compared to the HE group [41] at 3 months (MD 95% CI: 0.3 (−2.69 to 2.75)), at 6 months (MD 95% CI: −1.7 (−5.3 to 1.9)) and at 1 year (MD 95% CI: 0 (−3.2 to 3.2)), respectively, when measured with a 14-item modified version of the Shoulder Disability Questionnaire (SDQ) [51], associated with a rating scale on working ability.

Similarly, no differences were highlighted by comparison between the MUA group and SPT group [44] through Quick Disabilities of the Arm, Shoulder, and Hand (QuickDASH) [52] at 3-month follow-up (MD 95% CI: 1.77 (−3.41 to 6.96)), at 6-month follow-up (MD 95% CI: −3.55 (−8.68 to 1.58)) and at 1-year final follow-up (MD 95% CI: −0.50 (−5.70 to 4.70)), respectively.

#### 3.3.5. Other Outcomes

Health-related quality of life was lastly considered by two RCTs [43,44]. Using the five-level version of the EuroQoL 5-Dimension Questionnaire (EQ-5D-5L) [53], small to mild differences were highlighted between groups [44] in favor of the MUA group when compared to the SPT group, mostly at final 1-year follow-up, but without statically significant differences (MD 95% CI: 0.04 (−0.02 to 0.10)).

With similar results, all items of the Short-Form 36-Item Health Survey (SF-36) Questionnaire [54] improved in both MUA and SJHD groups [43], but no statistically significant differences were reported.

Lastly, one study [42] included the degree of patients’ satisfaction towards the received treatment, expressed with a modified three-level Likert scale (“very satisfied”, “satisfied” or “unsatisfied”). No statistical significances or clinical relevance were reported at the final follow-up of 6 months between groups.

#### 3.3.6. Adverse Events

Uncontrolled MUA procedures could trigger some adverse events, also reported as serious post-surgery complications [27,28], such as causing structural damages in the glenohumeral joint and its surrounding soft tissues. As described in one study [40], shoulder dislocation occurred in one case, while, in other patients, eventual capsular tears did not appear to be complicated by significant bleeding. Furthermore, when the MUA group was compared to the SPT group [44], two serious adverse events (1%) were reported in the MUA group. Non-serious events were also described in both groups, with similar rates (7% for MUA group and 5% for SPT group, respectively), and no evidence for statistical differences in the proportions between groups (*p* = 0.19) [44].

## 4. Discussion

The purpose of this SR was to investigate the efficacy and safety of MUA compared to other non-surgical treatment options for FSCS.

MUA was found to be not superior in terms of reduction of pain and improvement of function when compared to non-surgical strategies, both in the short term (1 and 3 months) [40,43] and in the long term (>6 months) [41,44]. Only one study [42] demonstrated that HD was superior to MUA in reducing pain and improving functional outcomes at 6 months. However, it is not possible to draw definitive conclusions, as confirmed by other systematic reviews [19,20]. A mild, clinically not relevant, yet statistically significant, difference was reported for anterior shoulder flexion in the short term (3 months) in favor of the MUA group, compared to HE [41]. As far as disability is concerned, statistically significant long-term (>12 months) differences [43,45,46] between groups (MUA vs. SJHD, HE and SPT, respectively) were not reported.

Due to the substantial heterogeneity of the studies included, mainly related to intervention types, control groups and outcome measures, the results obtained from the five eligible RCTs [40,41,42,43,44] should be interpreted with caution. Moreover, high RoB was reported in four [40,41,42,44] out of five of the studies included and small sample sizes were involved [40,42]. In addition, it was not possible to perform a quantitative synthesis (meta-analysis), so that evidence on the efficacy of MUA compared to conservative management can be hardly generalized.

Previous publications [10,28] confirmed that there is hardly any evidence regarding the superiority of MUA against conservative treatments. An SR on FSCS treatment options [10] declared that there was very little evidence available for MUA and most of the studies identified had several limitations, so that generalizability is somewhat unclear because of the limited information about previous interventions that participants had received and the appropriate stage of the pathology. Nevertheless, in another recent publication [28], the authors included indistinctly in their research both cohort and case–control studies, both retrospective and prospective. Observational studies are more prone to bias, often viewed with skepticism, and therefore their application remains contentious [55]. Recently, two other recent systematic reviews provided evidence that, when compared to each, neither physiotherapy techniques with a steroid injection nor MUA are clinically superior [24,56].

This SR questions some actual evidence on MUA procedures in its entirety. First of all, the adoption of different patient-related outcome measures related to function and disability [42,43,45,46] and the extensive variability of follow-up periods (from 4 weeks [40] up to 1 year [43,45,46]) do not allow us to reach definitive conclusions on the efficacy of MUA, both in the short and in the long term.

Although function is cited by healthcare professionals as, probably, the main outcome measure that should be used for shoulder disorders [57], the use of CS in two of the included RCTs [42,43] is questionable due to conflicting data with respect to its responsiveness [58,59]. Conversely, OSS, adopted in the most recent study [44], is a well-designed patient-reported measure of functional limitation following shoulder surgery. It is capable of detecting long-term changes [5] and its development and validation included patients with FSCS [47]. Moreover, other questionnaires, such as the Shoulder Pain and Disability Index (SPADI) [60] or Disabilities of the Arm, Shoulder, and Hand questionnaire (DASH) [61], were not considered, except for one study [44], and used as a secondary outcome to measure disability levels, despite being recommended in clinical guidelines [7] for better psychometric properties [62].

In addition, our SR highlighted the lack of interest in the subjective experience, emotional impact and patients’ priorities towards treatment [63]. Only one study reported the rate of satisfaction [42], which is extremely variable in other studies in the literature (from 30% [4] to 94% [25]). As suggested by the authors of a qualitative research work [6], a better understanding of patients’ experiences within a patient-centered healthcare paradigm could be useful for clinicians when evaluating the overall (and perceived) efficacy of the treatment [64] and should be considered by researchers [57].

Furthermore, two RCTs [43,44] included quality of life assessment at 1-year follow-up through the EQ-5D-5L questionnaire or SF-36, but no statistically significant differences between the two groups were found. According to this statement, preliminary core domain sets for shoulder pathology have been recently published, with the intent to identify the most appropriate outcomes to be measured, some of which are mandatory: pain, physical function/activity, global perceived effect and also adverse events [65].

The main disadvantage of the MUA procedure is that uncontrolled manipulation could cause structural damages in the glenohumeral joint and its surrounding soft tissues [32,66,67]. Even with a low complication rate of 0.4% [27], serious post-surgery complications are occasionally reported, especially when the procedure is performed with a long lever arm [10].

In our systematic review, only two RCTs [40,44] reported adverse events: one case of shoulder dislocation [40], one case of visual disturbance, headache, heaviness and numbness of arm and one case of septic arthritis [44]. Likewise, non-serious events were also described with similar rates, insufficient for formal analysis.

Nevertheless, it is interesting to point out slight differences in the types of non-serious events reported in both MUA and SPT groups (7% and 5%, respectively): injuries to adjacent structures of the manipulated shoulders, such as nerves, tendon, bone or joints, were reported, as well as neuropathic symptoms or pins and needles to the hand, postprocedural worsening of shoulder pain and persistent pain and/or stiffness requiring further treatment [44]. A recent publication [28] found an overall complication rate of 0.4% after MUA procedures and a re-intervention rate of 14%, though most of the included papers were not designed to monitor complications, as also happened in our SR. Thus, even if most iatrogenic capsular or other soft tissue lesions or bone edema detected with magnetic resonance imaging 1 week after MUA disappeared within 6 months and did not seem to be responsible for any clinical symptoms after this period [68], they should always be considered. Moreover, we could hypothesize that, due to its characteristics, MUA might also represent a disproportionate stimulus of tissue reaction, and therefore further fibrosis. As suggested in the past [69], more aggressive interventions, even when performed after one year of intensive physical treatment, did not change significantly the total duration of the disease, when compared to supervised neglect strategies. Moreover, a prospective study on a consecutive series of 792 patients with shoulder problems undergoing MUA [66] reported 714 patients (96%) who benefited from a single procedure (improvement in the OSS > 5 points), but of these, 53 patients (7%) had a relapse of FSCS within 3 months and another 45 (6%) had recurrence later (rate of 17.8% for a second procedure).

Similarly, one of the included studies [44] showed that following completion of their randomized treatment, some participants received further treatment. The further treatments were received by participants in the SPT group (*n* = 15 [15%]), followed by the MUA group (*n* = 14 [7%]), implying that such procedures could not be decisive, as often suggested. Hence, the enigma of such disease is perhaps not only the pathogenesis but the predictable effect of any therapy or treatment on the patients and the clinicians willing to give them fast relief.

As often reported, MUA is recommended to patients with FSCS that have poor results with physiotherapy [21,22]. However, patients’ characteristics regarding health status or previously received treatment were duly reported only in three out of five RCTs [43,45,46]. In this framework, some other authors [40,42] rely solely on the highly variable duration of symptoms, without considering essentials aspects of clinical history and clinical evaluation, such as comorbidities (e.g., endocrinopathies or diabetes), ongoing treatments or specific ROM restriction assessment, as suggested by Hanchard and colleagues [67]. A recent first retrospective case–control design study [70] analyzed the effectiveness of MUA for FSCS with comorbidities: the authors highlighted that ROM recovery speed and responsiveness to MUA were poorer for patients with comorbidities, mostly in the diabetes and thyroid disorder sub-groups. Thus, comorbidities can strongly affect the treatment results, suggesting that accurately diagnosing and identifying such factors could significantly affect treatment outcomes.

In the authors’ opinion, this aspect may represent a focus point for a core difference between MUA and other strategies. Among surgical regimens, MUA has been the long-standing treatment for refractory FSCS [71]: in fact, patient “resistant” to conservative treatments are more likely to be treated with MUA, and this could bias the baseline clinical presentation of pathology, which could be worse for the MUA group than in conservative strategy group patients. One could therefore suspect that only patients with more “resistant” FSCS had MUA. However, even if past published studies described a defined period of conservative management before proceeding to MUA intervention with a strong association between a delay in surgical management and poorer outcome [72], the most recent retrospective research [73] highlighted that there was no association between the improvement in outcome and the duration of presenting symptoms, thus confirming no concrete influence of the timing of MUA procedures in relation to short- and long-term outcomes in a cohort of patients with FSCS.

To date, the variable duration of the typically described phases in FSCS is not characterized by discrete periods of time but rather an evolution of symptoms and is difficult to measure individually. This is reflected in clinical practice when vague shoulder pain is only confidently diagnosed as FSCS once the presence of stiffness is strongly encountered. Thus, the rationale of a “resistant” or resolution phase as adequate for MUA choice of treatment seems to be inappropriate.

Moreover, it is still not clear if the patients that underwent MUA usually had to wait longer before receiving such treatment with respect to the conservative one. Unfortunately, only one study [44], with the largest sample size for the MUA group, highlighted a substantial difference between groups for treatment waiting time: participants waited a median of 14 days (IQR 7–22) for the SPT group and 57 days (IQR 35–89) for the MUA group. Studies that encompass an analysis of different waiting times for treatment should be therefore performed.

Moreover, this SR also highlights the significant heterogeneity in the management of MUA procedures (Table 3), confirming that FSCS management among orthopedic surgeons substantially varies based on personal experience, preferences and levels of confidence with treatment options, rather than relying on strong evidence [74].

As physiotherapists, we are partially heartened by the rehabilitation programs following MUA procedures shown in the RCTs [40,41,42,43,44]. However, the proposed educational strategies in four [40,41,42,43] out of five RCTs, albeit ethically essential, represent only a small part of the rehabilitation pathways; only one study [44], again, fully detailed a structured physiotherapy program (“hands on” manual therapy techniques [16] and exercise [18]) according to the stage of FSCS and accepted as good practice [67,75].

### 4.1. Strengths and Limitations

To the authors’ knowledge, this is the first SR conducted exclusively on RCTs comparing MUA to non-surgical treatments for the management of patients with FSCS. The protocol for this review was pre-registered in PROSPERO and followed PRISMA guidance for preparing a protocol and reporting a systematic review and meta-analysis [32,76]. Thus, a comprehensive and rigorous review was undertaken.

Therefore, the strengths of this SR are represented by the appropriate selection criteria, study design and included levels of evidence, as far as the adoption of the RoB 2.0 Cochrane tools.

Nevertheless, there are also several potential limitations in our study. The conclusions are based on the best available evidence’s synthesis, but it was not possible to avoid low-quality studies to minimize RoB. Despite accurate search strategies, even if comprehensive and based on the Population, Intervention, Comparison, Outcomes (PICO) approach, we may have missed some potentially relevant studies of interest. Studies written in languages other than those that were eligible for inclusion were excluded and, due to the heterogeneity of the included studies, it was not possible to perform a meta-analysis. Moreover, reference lists of potentially eligible articles in study registers (e.g., PROSPERO, ClinicalTrials.gov) were not screened.

Notably, our selection criteria did not allow us to include other published RCTs where MUA is frequently combined with other treatment procedures. It could be advisable to include these latter, so as to provide evidence of the effects (benefit or harm) of interventions that can feasibly be studied in RCTs, but for which only a small number of RCTs are available (or likely to be available) [34,35]. However, to the authors’ knowledge, very few “strong” non-randomized studies of interventions have been published, so that any further information regarding the efficacy of MUA could be provided.

### 4.2. Clinical Implications and Future Directions

Substantial ongoing research and future research is required to develop high-quality RCTs; these latter are needed to establish the benefits and harms of MUA interventions that reflect actual practice, also compared with placebos, no intervention and active interventions with evidence of benefit (e.g., steroid injection, at short term). In this direction, it would be interesting to reduce possible sources of between-study heterogeneity such as the phase of disease, presence of comorbidities and nuanced differences in treatment arms (e.g., steroid injection dose, physiotherapy approaches, standardized MUA procedures).

Future studies stratifying patients’ characteristics and nuances within treatment groups as independent contributors to clinical outcomes may provide additional knowledge to guide clinical decisions in the treatment of FSCS. Thus, adequate patient selection criteria and reliable assessment measures should also be considered.

Furthermore, patients’ perspectives on the experience of FSCS and their priorities for treatment, together with psycho-social aspects, are still marginally cited. Patient-reported outcomes measures, such as shoulder function and pain scores, together with clinical measurements such as shoulder range of motion, alongside complications or adverse events, are considered, but the authors only partially assess the impact of the condition on participants’ lives and their experience of such illness. In light of recent findings [77,78], a deeper assessment of psychological factors could be appropriate for the early recognition and management of these aspects that deeply impact patients’ conditions and, consequently, to better guide adequate treatment choices for FSCS.

Within a patient-centered healthcare paradigm, it is desirable that health professionals (physiotherapists and orthopedic surgeons in this circumstance) can soon find specific pathways for managing FSCS through an appropriate core domain set [65]. Thus, the evidence underpinning the management of FSCS is not strong and studies about the natural history of FSSC have produced somewhat contradictory results. Patients with shoulder disorders must face considerable disruption to their lives and FSCS remains undoubtedly a challenge for clinicians. Only through a complete and extensive understanding of its etiology and pathophysiology, as well as the identification of the best management strategies within a health economics framework, will it be possible to provide effective, patient-tailored interventions.

## 5. Conclusions

This review demonstrates that limited and uncertain evidence currently exists regarding the superiority of MUA versus conservative treatments in patients with FSCS. Few RCTs were included and small treatment effects in favor of MUA were observed due to limited evidence, which restricts the clinical relevance of findings; MUA as a stand-alone strategy to manage FSCS has not been studied with high-methodological-quality randomized controlled trials yet and with an appropriate sample size.

The authors therefore recommend extreme caution when MUA is suggested as a therapeutic option to reduce symptoms in FSCS: the quality of the available evidence is still inconclusive, collectively low and therefore unable to be generalized.

## Figures and Tables

**Table 1:** PICO components of the systematic review.

PICO	Features
Population	Patient suffering from frozen shoulder contracture syndrome (also called adhesive capsulitis)
Intervention	MUA (manipulation under anesthesia)
Comparator	Conservative treatment strategies (e.g., physical therapy, exercise, manual therapy, injection)
Outcome	Measures of pain, mobility, function, disability and quality of life

**Table 2:** Main characteristics of populations, interventions and outcome measures of included RCTs.

Author	Participants (*n*) Inclusion/Exclusion Criteria	Imaging Other Exams	Group of Intervention (GI)	Group of Control (GC)	Outcome Measures (S) Time of Follow-Up	Key Results
**Thomas** **et al.** [40]	30 (17 F, 15 M) Mean age (na) *Inclusion criteria:* ▪ primary FSCS confirmed by history and clinical findings ▪ spontaneous pain ▪ night pain ▪ movement limitations (na) *Exclusion criteria:* ▪ rotator cuff lesions ▪ supraspinatus tendonitis ▪ bicipital tendonitis ▪ sub-acromial bursitis ▪ inflammatory joint disease ▪ cervical spondylosis ▪ structural intrathoracic disease	X-ray (shoulder) X-ray (cervical spine) X-ray (thoracic spine) Blood picture, erythrocyte sedimentation rate, serum uric acid, random blood sugar	(na) Mean age 59.3 years (45–76) Mean symptom duration 7.4 months (2–24 months) **MUA GROUP** MUA with short general anesthesia (20 mg of intravenous valium and injection of 50 mg of hydrocortisone acetate, sub-acromial postero-lateral approach). Patients invited to perform exercises to maintain the improvement (self-induced passive stretching) and to manage residual pain with analgesics.	(na) Mean age 57.9 years (38–73) Mean symptom duration 7.4 months (2–24 months) **SJ GROUP** Short general anesthesia (20 mg of intravenous valium and injection of 50 mg of hydrocortisone acetate, sub-acromial postero-lateral approach). Patients invited to perform exercises to maintain the improvement (self-induced passive stretching).	**Pain** 4-point scale ^†^ (0–3) 4 weeks 3 months	**Pain (day)** At 3 months (*n* = 19) GI: *n* = 12 (80%) GC: *n* = 7 (47%) **Pain (night)** At 3 months (*n* = 19) GI: *n* = 12 (80%) GC: *n* = 7 (47%)
					**ROM** active 5-point scale ^#^ (0–4) 4 weeks 3 months	At 4 weeks, good response (*n* = 2) GI: *n* = 2 GC: *n* = 0 At 3 months, substantial recovery for 80% of FE and ABD (*n* = 8) GI: *n* = 6 (40%) GC: *n* = 2 (13%)
					**Disability** 4-point scale ^†^ (0–3) 4 weeks 3 months	At 4 weeks, recovered (*n* = 2) GI: *n* = 2 GC: *n* = 0 At 3 months, completely recovered (*n* = 9) GI: *n* = 7 (47%) GC: *n* = 2 (13%)
**Kivimäki** **et al.** [41]	125 (na) Mean age (na) *Inclusion criteria:* ▪ primary FSCS confirmed by history and clinical findings ▪ increasing pain ▪ decreasing joint mobility ▪ FE < 140° ▪ ER (arm at side) ≤ 30° ▪ allowed systemic disorders as diabetes mellitus (*n* = 18), hypertonia (*n* = 21) and asthma (*n* = 5) *Exclusion criteria:* ▪ traumatic events (bone or tendon changes) ▪ arthritis ▪ osteoarthritis ▪ rotator cuff lesion ore tears (suspected for weakness in ABD or ER movements)	US (rotator cuff)	65 Mean age 53.0 years (SD = 8.4) Mean symptom duration 7.4 months (SD = 0.3) **MUA GROUP** MUA with short general anesthesia. The patients received advice in 2 sessions and written instructions for a daily training program (pendulum exercises for the arm and stretching techniques for the shoulder joint) from a trained physical therapist.	65 Mean age 53.0 years (SD = 8.3) Mean symptom duration 7.0 months (SD = 0.3) **HE GROUP** Home exercise program. The patients received advice in 2 sessions and written instructions for a daily training program (pendulum exercises for the arm and stretching techniques for the shoulder joint) from a trained physical therapist.	**Pain** VAS ^‡^ (0–10) 6 weeks 3 months 6 months 1 year	At 6 weeks (4.9 GI vs. 4.7 GC) MD = 0.2 95% CI (−0.64 to 1.02) At 3 months (3.9 GI vs. 3.7 GC) MD = 0.2 95% CI (−1.06 to 1.10) At 6 months (2.0 GI vs. 2.8 GC) MD = −0.8 95% CI (−1.8 to 0.2) At 1 year (1.5 GI vs. 2.2 GC) MD = −0.7 95% CI (−1.8 to 0.4)
					ROM * passive goniometer ^§^ (°) 6 weeks 3 months 6 months 1 year	**Measures of FE:** At 6 weeks (133° GI vs. 129° GC) MD = 4° 95% CI (−3.8° to 11.8°) At 3 months (144° GI vs. 136° GC) MD = 8° (0° to 16°); *p* <0.05 At 6 months (151° GI vs. 146° GC) MD = 5° (−5° to 15°) At 1 year (157° GI vs. 154° GC) MD = 3° (−5° to 11°) **Measures of ABD:** At 6 weeks (125° GI vs. 112° GC) MD = 10° 95% CI (−3.2° to 23.2°) At 3 months (150° GI vs. 141° GC) MD = 9° (−6 to 24) At 6 months (151° GI vs. 142° GC) MD = 9° (−4° to 22°) At 1 year (161° GI vs. 154° GC) MD = 7° (−5° to 19°) **Measures of IR:** At 6 weeks (30° GI vs. 34° GC) MD = 4° 95% CI (−1° to 9°) At 3 months (22° GI vs. 25° GC) MD = −3° (−7.4° to 2.4°) At 6 months (16° GI vs. 18° GC) MD = −2° (−7.4° to 3.4°) At 1 year (11° GI vs. 12° GC) MD = −1° (−4.1° to 6.1°) **Measures of ER:** At 6 weeks (38° GI vs. 33° GC) MD = 5° 95% CI (−2° to 12°) At 3 months (48° GI vs. 42° GC) MD = 6° (−3° to 15°) At 6 months (59° GI vs. 53° GC) MD = 6° (−2° to 14°) At 1 year (65° GI vs. 61° GC) MD = 4° (−4.2° to 12.2°)
					**Disability** Modified SDQ ^††^ (0–28) 6 weeks 3 months 6 months 1 year Working ability ^‡‡^ (0–10) 6 weeks 3 months 6 months 1 year	**Disability (SDQ)** At 6 weeks (18.9 GI vs. 19.2 GC) MD = −0.3 95% CI (−2.3 to 1.7) At 3 months (14.5 GI vs. 14.2 GC) MD = 0.3 95% CI (−2.69 to 2.75) At 6 months (9.6 GI vs. 11.3 GC) MD = −1.7 95% CI (−5.3 to 1.9) At 1 year (6.6 GI vs. 6.6 GC) MD = 0 95% CI (−3.2 to 3.2) **Disability (Working ability)** At 6 weeks SDQ (6.6 GI vs. 6.2 GC) MD = −0.4 95% CI (−4.2 to 1.28) At 3 months SDQ (7.1 GI vs. 7.1 GC) MD = 0 95% CI (−0.8 to 0.8) At 6 months SDQ (7.8 GI vs. 7.3 GC) MD = 0.5 95% CI (−0.6 to 1.6) At 1 year SDQ (8.3 GI vs. 8.2 GC) MD = 0.1 95% CI (−0.8 to 1.0)
**Quraishi** **et al.** [42]	36 (21 F, 15 M) Mean age 55.2 y (39–70) *Inclusion criteria:* ▪ primary FSCS, stage II “freezing” ▪ global loss of active and passive ROM ▪ ER restriction (<50% opposite limb) *Exclusion criteria:* ▪ traumatic events or cause (na) ▪ extrinsic cause (na) ▪ suspected osteoporosis ▪ general anesthesia intolerance	X-ray (shoulder)	17 Mean age 54.5 years (39–69) Mean symptom duration 39.8 weeks **MUA GROUP** MUA with specific protocol and local anesthesia. Protocol to resume normal activities as soon as possible, home self-exercise program (pendular exercises and wall-climbing movements).	19 Mean age 55.2 years (44–70) Mean symptom duration 37.4 weeks **HD GROUP** Hydrodilatation by a consultant radiologist (anterior approach with radio-opaque contrast material and normal saline solution, 10 to 55 mL). Protocol to resume normal activities as soon as possible, home self-exercise program (pendular exercises and wall-climbing movements).	**Pain** VAS ^‡^ (0–10) 8 weeks 6 months	At 8 weeks GI: 4.7 95% CI (0.0 to 8.5) GC: 2.4 95% CI (0.0 to 8.0) At 6 months GI: 2.7 95% CI (0.0 to 9.0) GC: 1.7 95% CI (0.0 to 7.0) Between-group difference, *p* < 0.0001 in favor of hydrodilatation group compared to MUA group.
					**ROM** na (°) 8 weeks 6 months	Between-group difference in favor of hydrodilatation group compared to MUA group ABD: *p* < 0.0005 FE: *p* < 0.0004 IR: *p* = 0.02 ER: *p* = 0.004
					Function * CS ** (0–100) 8 weeks 6 months	At 8 weeks GI: 58.5 95% CI (24 to 90) GC: 57.4 95% CI (17 to 80) At 6 months GI: 59.5 95% CI (23 to 85) GC: 65.9 95% CI (28 to 92) Between-group difference, *p* = 0.02 in favor of hydrodilatation group compared to MUA group.
					**Satisfaction level** Modified Likert ^§§^ (0–2) 6 months	At 6 months, satisfied or very satisfied GI: 81% (*n* = 13) GC: 94% (*n* = 17)
**Jacobs** **et al.** [43]	53 (35 F, 18 M) Mean age (na) (40–75) *Inclusion criteria:* ▪ primary FSCS confirmed by history and clinical findings *Exclusion criteria:* ▪ type I and II diabetes ▪ previous steroid injections	X-ray (shoulder)	28 (15 F, 13 M) Mean age 56.5 years Mean symptom duration 19 weeks **MUA GROUP** MUA with general anesthesia (day-surgery treatment). Detailed brochure with home exercise delivered by physical therapists (unavailable description of dosage or type of provided home exercise program).	25 (20 F, 5 M) Mean age 57.0 years Mean symptom duration 16 weeks **SJHD GROUP** Injection with steroid and capsular distension, 3 treatments at 6-week intervals (40 mg of triamcinolone in 1 mL, 5 mL of 2% lignocaine, 10 mL of 0.25% bupivacaine and 5 mL of air, posterior route). Detailed brochure with home exercise delivered by physical therapists (unavailable description of dosage or type of provided home exercise program).	**Pain** VAS (0–100) 2 weeks 6 weeks 3 months 6 months 9 months 1 year	Main outcome measures subjected to regression analysis on the first 4 time points (change occurred in the first 16 weeks) and regression coefficients (of time) were compared, with no significant difference between treatment groups (95% CI (−1.11 to 1.15) GI: −2.77 (SE = 0.33) GC: −2.75 (SE = 0.42)
					**Function** CS ** (0–100) 2 weeks 6 weeks 3 months 6 months 9 months 1 year	Main outcome measures subjected to regression analysis on the first 4 time points (change occurred in the first 16 weeks) and regression coefficients (of time) were compared, with no significant difference between treatment groups (95% CI (−0.90 to 1.11) GI: 3.13 (SE = 0.24) GC: 3.23 (SE = 0.42)
					**Quality of life** SF-36 ^##^ (0–100) 1 year	All components of the SF-36 scores improved for all patients, but no statistically significant difference was found between groups.
**Rangan** **et al.** [44]	300 (na) Mean age (na) *Inclusion criteria:* ▪ unilateral FSCS confirmed by history and clinical findings ▪ passive ER restriction (<50% opposite limb) ▪ allowed diabetes (as significantly associated with impaired shoulder mobility) *Exclusion criteria:* ▪ bilateral concurrent FSCS ▪ traumatic events or cause (which require hospital care, e.g., locked posterior dislocation) ▪ secondary to other cause (e.g., breast surgery, glenohumeral arthritis) ▪ not having mental capacity to understand instruction or treatment ▪ not being a resident of catchment area of trial site (multicenter trial) ▪ any trial treatment contraindications (e.g., patient unfit for anesthesia or corticosteroid injection)	X-ray (shoulder)	201 (129 F, 72 M) Mean age 54.5 years (SD = 7.7) Mean symptom duration 10.5 months (SD = 8.6) **MUA GROUP** MUA with general anesthesia (day-surgery treatment). Provided post-surgery analgesia procedures (including nerve block procedures as per usual care). Intra-articular steroid injection of corticosteroid (with or without imaging guidance depending on usual practice of hospital site). Multiple (*n* = 12) sessions of structured physiotherapy over 12 w within 24 h: (a) focused physiotherapy (information leaflet containing education, advice on pain management and function, “hands-on” mobilization techniques, instruction on a graduated home exercise program progressing from gentle pendular exercises to firm stretching exercises); (b) supplemental physiotherapy (not essential intervention, but considered as permissible addition to allow some physiotherapists flexibility); (c) intra-articular steroid injection of corticosteroid. Treatment features were selected according to potential different stages of FSCS, as stated in systematic reviews, UK guidelines and previous surveys of UK physiotherapists and Delphi consensus methodology.	99 (64 F, 35 M) Mean age 54.5 years (SD = 7.8) Mean symptom duration 10.8 months (SD = 8.8) **SPT GROUP** Multiple (*n* = 12) sessions of structured physiotherapy over 12 w: (a) focused physiotherapy (information leaflet containing education, advice on pain management and function, “hands-on” mobilization techniques, instruction on a graduated home exercise program progressing from gentle pendular exercises to firm stretching exercises); (b) supplemental physiotherapy (not essential intervention, but considered as permissible addition to allow some physiotherapists flexibility); (c) intra-articular steroid injection of corticosteroid. Treatment features were selected according to potential different stages of FSCS, as stated in systematic reviews, UK guidelines and previous surveys of UK physiotherapists and Delphi consensus methodology.	**Pain** NRS ^‡^ (0–10) 3 months 6 months 9 months 1 year	At 3 months (4.1 GI vs. 3.7 GC) MD = 0.43 95% CI (−0.17 to 1.03); *p* = 016 At 6 months (2.8 GI vs. 3.0 GC) MD = −0.19 95% CI (−0.78 to 0.40); *p* = 0.53 At 1 year (2.4 GI vs. 2.5 GC) MD = −0.08 95% CI (−0.66 to 0.50); *p* = 0.78
					**Function** OSS *^,^ *** (0–48) 3 months 6 months 9 months 1 year	At 3 months (30.2 GI vs. 31.6 GC) MD = −1.36 95% CI (−3.70 to 0.98); *p* = 0.25 At 6 months (37.1 GI vs. 34.9 GC) MD = 2.15 95% CI (−0.12 to 4.42); *p* = 0.064 At 1 year (38.3 GI vs. 37.2 GC) MD = 1.05 95% CI (−1.28 to 3.39); *p* = 0.38 Median overall OSS of 43 (out of 48) points, compared with an initial median overall OSS of 20 points for both groups.
					**Disability** QuickDASH ^†††^ (0–100) 3 months 6 months 9 months 1 year	At 3 months (38.8 GI vs. 37.1 GC) MD = 1.77 95% CI (−3.41 to 6.96); *p* = 0.50 At 6 months (27.7 GI vs. 29.2 GC) MD = −3.55 95% CI (−8.68 to 1.58); *p* = 0.18 At 1 year (29.9 GI vs. 23.4 GC) MD = −0.50 95% CI (−5.70 to 4.70); *p* = 0.85
					**Quality of life** EQ-5D-5L ^###^ (0–5) 3 months 6 months 9 months 1 year	At 3 months (0.63 GI vs. 0.61 GC) MD = 0.03 95% CI (−0.03 to 0.08); *p* = 0.38 At 6 months (0.73 GI vs. 0.68 GC) MD = 0.05 95% CI (−0.01 to 0.10); *p* = 0.10 At 1 year (0.73 GI vs. 0.69 GC) MD = 0.04 95% CI (−0.02 to 0.10); *p* = 0.20
					**Extent of recovery** VAS (0–100) ^§§§^ 3 months 6 months 9 months 1 year	**Extent of recovery** At 3 months (51.54 GI vs. 53.9 GC) MD = −2.55 95% CI (−11.68 to 6.58); *p* = 0.58 At 6 months (31.9 GI vs. 38.6 GC) MD = −6.71 95% CI (−15.83 to 2.42); *p* = 0.15 At 1 year (27.3 GI vs. 26.9 GC) MD = 0.46 95% CI (−7.79 to 8.70); *p* = 0.91
					**Economic analysis** QALYs Over 1 year	The base-case health economic analysis with multiple imputation showed that MUA was GBP 276.51 (95% CI 65.67 to 487.35) more expensive per participant than was early structured physiotherapy. MUA was the intervention most likely to be cost-effective at a threshold of GBP 20,000 per QALY (GI = 86%; GC = 14%).

* Reported as primary outcome. **^†^** Scored as “0” if worse, “1” if no change, “2” if improved and “3” if cured. **^‡^** Scored on an 11-point scale where 0 represents no pain at all and 10 is unbearable pain. **^#^** Scored as “0” if worse, “1” if no change, “2” if slight, “3” if moderate, “4” if good and “5” if cured. **^§^** Measured for forward flexion, abduction, external and internal rotation. ** Divided into four subscales: pain (15 points), activities of daily living (20 points), strength (25 points) and range of motion (40 points). **^††^** Pain evaluated in 14 activities of daily living during previous 24 h, with “perceived pain” receiving 2 points, “cannot say” receiving 1 point and “no pain” receiving 0 points. **^‡‡^** Scored on an 11-point scale where 0 represents total inability to work and 10 indicates work ability at its best. **^##^** Multi-item scale that assesses 8 health concepts (vitality, physical functioning, bodily pain, general health perceptions, physical role functioning, emotional role functioning, social role functioning and general mental health); each scale is directly transformed into a 0–100-point scale on the assumption that each question carries equal weight. **^§§^** Scored as “0” if dissatisfied, “1” if satisfied, “2” if very satisfied. *** Modified version with 12 items, scored from 4 (best/fewest symptoms) to 0 (worst/most severe), with a lower score indicating a greater degree of disability. **^†††^** Subset of 11 items from the original version of 30-item DASH; presented as 5-point Likert scales, at least 10 of the 11 items must be completed for a score to be calculated and the scores range from 0 (no disability) to 100 (most severe disability). **^###^** Heath measure using three levels of severity in five dimensions and a new 5-level version to increase reliability and sensitivity (discriminatory power) of the scale; scored from “no problems” to “unable to/extreme problems”. **^§§§^** Ranging from 0 (“no need to seek further treatment”) to 100 (“definite need”). **Acronyms:** ABD: abduction; CS: Constant–Murley Shoulder Function Assessment Score; CI: confidence interval; ER: external rotation; EQ-5D-5L: EuroQoL 5-Dimension Questionnaire; F: female; FE: forward flexion; FSCS: Frozen Shoulder Contracture Syndrome; GC: group of control, GI: group of intervention; HD: hydrodilatation; HE: home exercise; M: male; mg: milligram; MD: mean difference; MUA: manipulation under anesthesia; na: not available; *p*: *p*-value; QALYs: quality-adjusted life years; QuickDASH: Quick Disabilitiy of the Arm, Shoulder and Hand Questionnaire; ROM: range of motion; SD: standard deviation; SDQ: Shoulder Disability Questionnaire; SE: standard error; SF-36: Short-Form 36-Item Health Survey Questionnaire; SJ: steroid and anesthetic intra-articular injection; SJHD: steroid and anesthetic intra-articular injection associated with hydrodilatation; SPT: steroid and early structured physiotherapy; VAS: visual analogue scale; vs: versus.

**Table 3:** Characteristics of MUA procedures and reported adverse events.

Author	Type of Anesthesia	Involved Operator	Sequence of Manipulation	Additional Precautions	Adverse Events/Complications
**Thomas** **et al**. [40]	Short general (intravenous 20 mg Valium, 50 mg hydrocortisone acetate)	1	90° ABD (forced) IR ER	(na)	*n* = 1 (GH dislocation)
**Kivimäki** **et al.** [41]	Short general	1	FE ABD IR (gm) ER (gm)	Supine patient Scapular stabilization	(na)
**Quraishi** **et al.** [42]	Local (2 mL al 2.00% lignocaine, 30 mg (0.75 mL) triamcinolone acetonide)	1	(na)	Short lever	(na)
**Jacobs** **et al**. [43]	General	2	ADD FE IR ER ABD	Supine patient Scapular stabilization Short lever	(na)
**Rangan** **et al**. [44]	General	(na)	(na)	(na) Additional steroid injection	*n* = 2 (visual disturbances, headache, numbness, heaviness of the arm; septic joint arthritis)

**Acronyms:** ADD: adduction; ABD: abduction; ER: external rotation; IR: internal rotation; FE: forward flexion; GH: glenohumeral; gm: gentle maneuver; mg: milligram; mL: millimeter; na: not available.

## Data Availability

Data is contained within the article or Supplementary Materials.

## Supplementary Materials

The following supporting information can be downloaded at: https://www.mdpi.com/article/10.3390/ijerph19159715/s1, Table S1: Search strategies; Table S2: RCTs excluded with reason; Table S3: Others excluded studies, with reason; Table S4: PRISMA Checklist for reporting.
