# Supplementary material for: Manipulation under Anesthesia versus Non-Surgical Treatment for Patients with Frozen Shoulder Contracture Syndrome: A Systematic Review
Source: Int J Environ Res Public Health. 2022 Aug 7;19(15):9715. doi: 10.3390/ijerph19159715 (PMC9368476; doi:10.3390/ijerph19159715)
Supplement: Supplementary file 1 [file ijerph-19-09715-s001.zip › IJERPH _ Table S1.pdf]

Supplementary materials

Table S1. Search strategies.

Database	Strategies	Notes
**MEDLINE** (PubMed)	((((((("Bursitis"[Mesh] OR "Periarthritis"[Mesh]) OR "Dupuytren Contracture"[Mesh]) OR ("bursitis"[MeSH Terms] OR "bursitis"[All Fields] OR ("frozen"[All Fields] AND "shoulder"[All Fields]) OR "frozen shoulder"[All Fields])) OR ("bursitis"[MeSH Terms] OR "bursitis"[All Fields] OR ("adhesive"[All Fields] AND "capsulitis"[All Fields]) OR "adhesive capsulitis"[All Fields])) OR (stiff[All Fields] AND ("shoulder"[MeSH Terms] OR "shoulder"[All Fields]))) OR (("bursitis"[MeSH Terms] OR "bursitis"[All Fields] OR ("frozen"[All Fields] AND "shoulder"[All Fields]) OR "frozen shoulder"[All Fields]) AND ("contracture"[MeSH Terms] OR "contracture"[All Fields]) AND ("syndrome"[MeSH Terms] OR "syndrome"[All Fields])))) AND (((((((((anaesthesia[All Fields]) OR manipulation[All Fields])) OR ((manipulation[All Fields] AND under[All Fields] AND ("anaesthesia"[All Fields] OR "anesthesia"[MeSH Terms] OR "anesthesia"[All Fields])) OR MUA[All Fields])) OR MUA[All Fields]) OR "Neuromuscular Blockade"[Mesh]) OR "Musculoskeletal Manipulations"[Mesh]) OR "Manipulation, Orthopedic"[Mesh]) OR ("anaesthesia"[All Fields] OR "anesthesia"[MeSH Terms] OR "anesthesia"[All Fields]))	Search filters:<br>▪ Publication date: from inception until 31st January 2022<br>▪ Language: English, German, Italian
**Embase**	('frozen shoulder' OR 'humeroscapular periarthritis' OR bursitis) AND ('manipulative medicine' OR 'manipulation under anesthesia')	
**PEDro**	▪ Title and abstract: frozen shoulder manipulation<br>▪ Title and abstract: adhesive capsulitis manipulation<br>▪ Title and abstract: frozen shoulder manipulation under anesthesia<br>▪ Title and abstract: adhesive capsulitis manipulation under anesthesia<br>▪ Title and abstract: frozen shoulder MUA<br>▪ Title and abstract: adhesive capsulitis MUA	The results of the 6 search strategies are combined.
**Cochrane Library**	(frozen shoulder OR adhesive capsulitis OR bursitis) AND (manipulation OR MUA)	
**Scopus**	TITLE-ABS KEY ((periarthritis OR (dupuytren AND contracture) OR bursitis OR (adhesive AND capsulitis) OR (frozen AND shoulder) OR (stiff AND shoulder)) AND (manipulation OR (manipulation AND under AND anaesthesia) OR mua))	Search filters:<br>▪ Publication date: from inception until until 31st January 2022<br>▪ Language: English, German, Italian
