# Supplementary material for: Manipulation under Anesthesia versus Non-Surgical Treatment for Patients with Frozen Shoulder Contracture Syndrome: A Systematic Review
Source: Int J Environ Res Public Health. 2022 Aug 7;19(15):9715. doi: 10.3390/ijerph19159715 (PMC9368476; doi:10.3390/ijerph19159715)
Supplement: Supplementary file 1 [file ijerph-19-09715-s001.zip › IJERPH _ Table S2.pdf]

Supplementary materials

Table S2. RCTs excluded with reason.

Author, year	Title, journal	Reason for exclusion	Intervention group	Control group
Amir-Us-Saqlain et al 2007	Functional outcome of frozen shoulder after manipulation under anaesthesia. *J Pak Med Assoc 57(4): 181-185.*	Different comparison	Manipulation under anesthesia Steroid injection Extremity kept in ABD and ER	Manipulation under anesthesia Steroid injection
Atalay et al 2021	Clinical effects of suprascapular nerve block in addition to intra-articular corticosteroid injection in the early stages of adhesive capsulitis: A singleblind, randomized controlled trial. *Acta Orthop Traumatol Turc* 2021; 55(6): 459-465	Different comparison	Scapular nerve block procedures Steroid injection	Steroid injection
Hussainet et al 2018	Outcome of manipulation under anesthesia in treatment of frozen shoulder with and without steroid injection in terms of range of motion. *Medical Forum Monthly 29(2): 79-83.*	Different comparison	Manipulation under anesthesia Steroid injection	Manipulation under anesthesia
Jones et al 2000	Suprascapular nerve block for the treatment of frozen shoulder in primary care: a randomized trial. *Br J Gen Pract 49(438): 39-41.*	Different intervention features	Scapular nerve block procedures	Steroid injection
Karatas et al 2002	Suprascapular nerve block for pain relief in adhesive capsulitis: comparison of 2 different techniques. *Arch Phys Med Rehabil 83(5): 593-597.*	Different intervention features	Scapular nerve block procedures (needle tip guided)	Scapular nerve block procedures (near-nerve electromiographially guided)
Kivimäki et al 2001	Manipulation under anesthesia for frozen shoulder with and without steroid injection. *Arch Phys Med Rehabil 82(9): 1188-1190.*	Different comparison	Manipulation under anesthesia Steroid injection	Manipulation under anesthesia
Kivimäki et al 2002	The effectiveness of manipulation under anesthesia for the frozen shoulder. Randomized controlled trial. *(na)*	Different language (other language: Finnish)	Manipulation under anesthesia	Physical therapy exercise advice
