# Supplementary material for: Manipulation under Anesthesia versus Non-Surgical Treatment for Patients with Frozen Shoulder Contracture Syndrome: A Systematic Review
Source: Int J Environ Res Public Health. 2022 Aug 7;19(15):9715. doi: 10.3390/ijerph19159715 (PMC9368476; doi:10.3390/ijerph19159715)
Supplement: Supplementary file 1 [file ijerph-19-09715-s001.zip › IJERPH _ Table S3.pdf]

Supplementary materials

Table S3. Other excluded studies with reason.

Author, year	Title, journal	Reason for exclusion
Ahmad et al. 2014	Outcome of manipulation under anaesthesia in adhesive capsulitis patients. *J Coll Physicians Surg Pak 24(4): 293-294.*	Different study design (case series)
Andersen et al. 1998	Frozen shoulder: arthroscopy and manipulation under general anesthesia and early passive motion. *J Shoulder Elbow Surg 7(3): 218-222.*	Different study design (case series)
Ando et al. 2018	Short-term Clinical Results of Manipulation Under Ultrasound-Guided Brachial Plexus Block in Patients with Idiopathic Frozen Shoulder and Diabetic Secondary Frozen Shoulder. *Open Orthop J 12: 99-104.*	Different intervention/comparison features
Dodenhoff et al. 2000	Manipulation under anesthesia for primary frozen shoulder: effect on early recovery and return to activity. *J Shoulder Elbow Surg 9(1): 23-26.*	Different study design (case series)
Fahmy et al. 2018	Under Anesthesia Thaws Frozen Shoulder. J Am Osteopath Assoc 118(7): 485-486.	Different study design (clinical commentary)
Farrell et al. 2005	Manipulation for frozen shoulder: long-term results. *J Shoulder Elbow Surg 14(5): 480-484.*	Insufficient available data
Flannery et al. 2007	Adhesive shoulder capsulitis: does the timing of manipulation influence outcome? *Acta Orthop Belg 73(1): 21-25.*	Insufficient available data
Ghosh et al. (na)	Comparison of results of three different methods of treatment for adhesive capsulitis of shoulder. *J Indian Med Assoc 110(11): 827-828.*	Full-text not available after author's request
Hamdan et al. 2003	Manipulation under anaesthesia for the treatment of frozen shoulder. *Int Orthop 27(2): 107-109.*	Different study design (case series)
Helbig et al.	Mobilization of frozen shoulder under general anaesthesia. *Acta Orthop Belg 49(1): 267-274.*	Different study design (case series)
Hsu et al. 1991	Arthroscopic distension in the management of frozen shoulder *Int Orthop 15(2): 79-83.*	Different study design (case series)
Jacchia et al.	[Mobilization under anesthesia in periarthritis of the shoulder].	Different study design

1968	*Arch Putti Chir Organi Mov 23: 231-241.*	(book chapter)
Jacobs et al.<br>2005	Comparision of manipulation under anaesthetic and distension injections in the management of frozen shoulder.	Different study design<br>(meeting abstract)
Jenkins et al.<br>2012	The outcome of manipulation under general anesthesia for the management of frozen shoulder in patients with diabetes mellitus.<br>*J Shoulder Elbow Surg 21(11): 1492-1498.*	Different intervention/comparison features
Khan et al.<br>2009	Manipulation under local anesthesia in idiopathic frozen shoulder--a new effective and simple technique.<br>*Nepal Med Coll J 11(4): 247-253.*	Insufficient available data
Kraal et al.<br>2019	Manipulation under anesthesia for frozen shoulders: a retrospective cohort study.<br>*Acta orthopaedical Belgica 85(4):400-405.*	Different study design<br>(case series)
Loyd et al.<br>1983	Adhesive capsulitis of the shoulder: arthrographic diagnosis and treatment.<br>*South Med J 76(7): 879-883.*	Different study scope
Mahmood et al.<br>2018	Outcome of manipulation under anesthesia in frozen shoulder with and without steroid injection in terms of range of motion.<br>*Pakistan Journal of Medical and Health Sciences 12(4): 1699-1702.*	Kinship
McKean et al.<br>2018	Shoulder manipulation under targeted ultrasound-guided rotator interval block for adhesive capsulitis.<br>*Skeletal Radiol 48(8): 1269-1274.*	Different intervention/comparison features
Melzer et al.<br>1995	Frozen shoulder--treatment and results.<br>*Arch Orthop Trauma Surg 114(2): 87-91.*	Different study design<br>(case series)
Meyer at al.<br>2015	[Management of Idiopathic Frozen Shoulder - Prospective Evaluation of Mobilisation under Anaesthesia and an Additional Subacromial Cor-<br>tisone Injection].<br>*Z Orthop Unfall 153(6): 613-617.*	Different intervention/comparison features
Ng et al.<br>2009	Manipulation under anaesthesia and early physiotherapy facilitate recovery of patients with frozen shoulder syndrome.<br>*Scott Med J 54(1): 29-31.*	Insufficient available data
Omari et al.<br>2001	Open surgical release for frozen shoulder: surgical findings and results of the release.<br>*J Shoulder Elbow Surg 10(4): 353-357.*	Different intervention/comparison features
Othman et al.<br>2002	Manipulation under anaesthesia for frozen shoulder.<br>*Int Orthop 26(5): 268-270.*	Insufficient available data
Pap et al.<br>1998	[Results of mobilization under anesthesia in adhesive capsulitis in relation to stage of the disease].<br>*Z Orthop Ihre Grenzgeb 136(1): 13-17.*	Insufficient available data
Placzek et al.<br>1998	Long-term effectiveness of translational manipulation for adhesive capsulitis.<br>*Clin Orthop Relat Res(356): 181-191.*	Different intervention/comparison features
Quraishi et al.<br>2008	Thawing the frozen shoulder - a randomised controlled trial of manipulation under anaesthetic versus hydrodilatation.	Different study design<br>(meeting abstract)
Reichmister et al.		Full-text not available after author's request

**(na)**	Long-term functional results after manipulation of the frozen shoulder.<br>*Md Med J 48(1): 7-11.*	
**Rendeiro et al.<br>2019**	Effectiveness of translational manipulation under interscalene block for the treatment of adhesive capsulitis of the shoulder.<br>A nonrandomized clinical trial.<br>*Physiother Theory Pract 35(8): 703-723.*	Different intervention/comparison features
**Rill et al.<br>2011**	Predictors of outcome after nonoperative and operative treatment of adhesive capsulitis.<br>*Am J Sports Med 39(3): 567-574.*	Different study scope
**Sasanuma et al.<br>2016**	Magnetic resonance imaging and short-term clinical results of severe frozen shoulder treated with manipulation under ultrasound-guided cervical nerve root block.<br>*Journal of Shoulder and Elbow Surgery 25(1): e13-e20.*	Different study design<br>(case series)
**Sharma et al.<br>1993**	Frozen shoulder syndrome. A comparison of hydraulic distension and manipulation.<br>*Int Orthop 17(5): 275-278.*	Different intervention/comparison features
**Singh et al.<br>(na)**	Manipulation under anaesthesia for treatment of adhesive capsulitis with early physiotherapy - A pilot study.<br>*JMS - Journal of Medical Society 24(1): 12-15.*	Full-text not available after author's request
**Song et al.<br>2021**	Outcome of manipulation under anesthesia with or without intra-articular steroid injection for treating frozen shoulder: A retrospective co-hort study.<br>*Medicine (Baltimore) 100(13):e23893.*	Different study design<br>(case series)
**Sokk et al.<br>2012**	Recovery of shoulder muscle function characteristics and active range of motion in patients with frozen shoulder after manipulation under anesthesia.<br>*European Orthopaedics and Traumatology 3(3): 169-176.*	Insufficient available data
**Sokk et al.<br>2012**	Shoulder muscle isometric strength and active range of motion in patients with frozen shoulder syndrome after manipulation under anes-thesia.<br>*Medicina (Lithuania) 48(7): 331-337.*	Insufficient available data
**Sokk et al.<br>2013**	Shoulder muscle function in frozen shoulder syndrome patients following manipulation under anesthesia: A 6-month follow-up study.<br>*Orthopaedics and Traumatology: Surgery and Research 99(6): 699-705.*	Different study scope
**Takahashi et al.<br>2020**	Clinical results of shoulder manipulation under ultrasound-guided cervical nerve root block for frozen shoulder in patients with diabetes.<br>*Journal of orthopaedics: 297-301.*	Different intervention/comparison features
**Theodorides et al.<br>2012**	Factors affecting short- and long-term outcomes of manipulation under anaesthesia in patients with adhesive capsulitis of the shoulder.<br>*Shoulder Elbow 6(4): 245-256.*	Different study scope
**Thomas et al.<br>2011**	Treatment of frozen shoulder by manipulation under anaesthetic and injection: does the timing of treatment affect the outcome?<br>*J Bone Joint Surg Br 93(10): 1377-1381.*	Different study scope
**Tsvieli et al.<br>2017**	Manipulation under anaesthetic for frozen shoulder using Codman's paradox: a safe and early return of function.<br>*Int Orthop 42(2): 339-344.*	Insufficient available data
**Van Royen et al.<br>1996**	Treatment of frozen shoulder by distension and manipulation under local anaesthesia.<br>*Int Orthop 20(4): 207-210.*	Different intervention/comparison features

**Vastamaki et al. 2012**	The natural history of idiopathic frozen shoulder: a 2- to 27-year followup study. *Clin Orthop Relat Res 470(4): 1133-1143.*	Critical risk of bias after ROBINS-I assessment for NRSI*
**Vastamaki et al. 2013**	Optimal time for manipulation of frozen shoulder may be between 6 and 9 months. *Scand J Surg 104(4): 260-266.*	Insufficient available data
**Vastamaki et al. 2015**	Motion and pain relief remain 23 years after manipulation under anesthesia for frozen shoulder. *Clin Orthop Relat Res 471(4): 1245-1250.*	Kinship
**Wallny et al. 1997**	["Primary" shoulder stiffness: illness duration and therapeutic comparison]. *Z Orthop Ihre Grenzgeb 135(3): 222-227.*	Different study scope
**Wang et al. 2007**	Comparison of idiopathic, post-trauma and post-surgery frozen shoulder after manipulation under anesthesia. *Int Orthop 31(3): 333-337.*	Different intervention/comparison features
**Wang et al. 2010**	Manipulation under anaesthesia for frozen shoulder in patients with and without non-insulin dependent diabetes mellitus. *International Orthopaedics 34(8): 1227-1232.*	Kinship
**Weber at al. 1995**	Long-term follow up to patients with frozen shoulder after mobilization under anesthesia, with special reference to the rotator cuff. *Clin Rheumatol 14(6): 686-691.*	Different study scope
**Weiser et al. 1977**	Painful primary frozen shoulder mobilization under local anesthesia. *Arch Phys Med Rehabil 58(9): 406-408.*	Different study design (case series)
**Wiley et al. 1991**	Arthroscopic appearance of frozen shoulder. *Arthroscopy 7(2): 138-143.*	Different study scope
**Woods et al. 2017**	Recurrence of frozen shoulder after manipulation under anaesthetic (MUA): the results of repeating the MUA. *Bone Joint J 99(6): 812-817.*	Different intervention/comparison features
**Zhang et al. 2020**	Back arm flexion and extension combined with manipulation for the treatment of patients with adhesive scapulohumeral periarthritis. *China Journal of orthopaedics and traumatology 33(7): 662-664.*	Different study design (case series)
